# Supplementary material for: Maternal and newborn healthcare providers’ work-related experiences during the COVID-19 pandemic, and their physical, psychological, and economic impacts: Findings from a global online survey
Source: PLOS Glob Public Health. 2022 Aug 5;2(8):e0000602. doi: 10.1371/journal.pgph.0000602 (PMC10021724; doi:10.1371/journal.pgph.0000602)
Supplement: S1 Appendix — (DOCX) [file pgph.0000602.s001.docx]

**S1 Appendix – Survey questions included in the analysis**

**Preparedness and response to COVID-19: a global survey of maternal and newborn care providers**

**Round 2 questionnaire**

**We would like to ask a few questions about your background**

| **Question** | **Response** |
| --- | --- |
| In which **country** are you based (providing healthcare) at the moment? | [drop down menu of countries] |
| What is your main **job**?  (choose one) | Midwife  Nurse-midwife  Nurse  Obstetrician/gynaecologist  Neonatologist  Paediatrician  Medical Doctor  Other (Please specify other job) |
| What is your **position**?  (choose one) | Head of facility (director, administrator)  Head of department or ward  Head of team  Team member  Locum or interim member  Independent or self-practicing  Other (Please specify other position) |
| What is your **gender**?  (choose one) | Female  Male  Other/Prefer not to say |
| What **type of maternal and/or neonatal health care** do you currently provide as an individual?  (select all that apply) | Antenatal care  Intrapartum care  Postnatal care  Outpatient breastfeeding support  Neonatal care for small and sick newborns  Surgical care  Family planning provision or counselling  Abortion and post-abortion care  Home care or community outreach  Other (Please specify other care type) |

**Setting: Can you tell us about the setting in which you work now?**

| **Question** | **Response** |
| --- | --- |
| Do you work in more than one health facility or for more than one employer? | Yes  No |
| In which **level** of health care institution do **you primarily work**?  Select the one where you spend most of your time.  (if none of the response options fit well, please use the “Other” option and write what your facility type is called in your country) | Referral hospital  District/regional hospital  Health centre  Polyclinic or Clinic  Birth centre  Health post/unit  Dispensary  Home-based care  Independent/self-practicing  Other (Please specify) |
| What organisation **type** is your **primary** institution or employer?  (if none of the response options fit well, please use the “Other” option and write what your organisation type is called in your country) | Public (national)  Public (university or teaching)  Public (district level or lower)  Social security  Health insurance  Private university  Private not-for-profit  Private for profit  Non-governmental  Faith-based or mission  Self-practicing  Other (Please specify) |
| In what type of **geographic area** do you provide care at the moment? | Large city (>1 mil inhabitants)  Small city (100,000 to 1 mil inhabitants)  Town (<100,000 inhabitants)  Village or rural area  Refugee or displaced persons camp  Other (Please specify) |

**Response to COVID-19 at this point in time**

| **Question** | **Response** |
| --- | --- |
| At this time, is it possible for you as a health worker to be tested for COVID-19?  (RT-PCR test, which detects the presence of the SARS-CoV-2 virus) | - Yes – regardless of symptoms or exposure - Yes – only if exposed to COVID-19 suspected/confirmed cases - Yes – only if symptomatic - No - Don’t know |
| Do you, as a health worker, have to pay for this test?  (If answer to previous question = Yes) | Yes  No, it is free of charge  Don’t know  Other (please specify) |
| On a scale from 1 (not at all) to 5 (completely), do you feel that you are sufficiently protected from infection with COVID-19 in your workplace? | 1 – not at all  2 – minimal protection  3 – some protection  4 – well protected  5 – completely protected |
| At this time, is **sufficient quantity** of the following types of personal protective equipment (PPE) available **to protect you?** |  |
| Gloves | Yes – No – Not-required |
| N-95 or FFP2 face masks | Yes – No – Not-required |
| Surgical face masks | Yes – No – Not-required |
| Face shields, goggles or eye protection | Yes – No – Not-required |
| Aprons | Yes – No – Not-required |
| Is personal protective equipment available to you in sufficient quantity **to change between patients**? | Yes  No |
| At this time, are you experiencing any challenges related to PPE availability/use and the provision of care to women and newborns? | Yes  No |

**Your work and experience in light of the COVID-19 outbreak**

| **Question** | **Response** |
| --- | --- |
| At this time, how easy is it for you to **reach** your workplace on a daily basis?  Either facility or patients’ homes | - I can reach my workplace easily - I can reach my workplace with some difficulty - It is very difficult for me to reach the workplace - It is impossible for me to reach the workplace |
| Why is it difficult for you to reach the workplace?  Select all that apply  (If answer to previous question is not “easily”) | - Lockdown measures - Curfew or quarantine - Public transportation availability - Other (please specify) |
| During the past month, were there any changes in staffing levels in your ward or practice? | -Yes, staffing levels decreased  -Yes, staffing levels increased  -No, staffing not affected  -Don’t know |
| Please specify why staffing level decreased  (select all that apply)  (If answer to previous question is “decreased”) | - Change in staff rotation or shift schedule - Staff unable to reach workplace - Staff re-assigned to COVID-19 wards - Staff isolating following exposure to COVID-19 - Staff ill with COVID-19 - Staff off due to childcare - Staff off due to stress or burnout - Don’t know - Other (please specify) |
| Compared to your income before the COVID-19 pandemic, is your current income? | Substantially higher  Somewhat higher  About the same  Somewhat lower  Substantially lower  Don’t know |
| In the past month, were you exposed to aggressive behavior while at work or related to your job as a healthcare professional as a result of the COVID-19 pandemic?  This includes any verbal, nonverbal or physical behavior that was threatening, or physical behavior that caused harm - to you, others (colleagues/family members/friends) or to property. | Yes  No |
| What was the type of behaviour that you were exposed to?  (select all that apply, if multiple incidents, include all in the past month)  (If was exposed to aggressive behaviour) | - Animosity or discrimination - Harassment - Verbal aggression, shouting - Intimidation / threats - Threatening gestures, including with a weapon or a dangerous object (syringe etc) - Physical violence (including shoving, punching, kicking, biting, scratching, strangling, throwing objects, etc) - Spitting or coughing - Sexual violence - Self-harm - Other (please specify) |
| Who was the target of this behaviour?  (select all that apply, if multiple incidents, include all in the past month)  (If was exposed to aggressive behaviour) | - Myself - My colleagues - My family members - My friends or relatives - Patients - Aggression toward objects (desk, wall, etc) - Other (please specify) |
| Who was the perpetrator of this behaviour?  (select all that apply, if multiple incidents, include all in the past month)  (If was exposed to aggressive behaviour) | - Myself - My colleague(s) - My family member(s) - My friend(s) or relative(s) - Someone from my community (for example, neighbour or teacher) - Patient - Patient’s family - Stranger - Public or government official - Other (please specify) |
| Compared to the beginning of the COVID-19 outbreak, how would you rate your own levels of stress in the past month? | - Substantially lower - Somewhat lower - Same as the beginning of the outbreak - Somewhat higher - Substantially higher |
| Do you have access to formal mental and psychological support? | - Yes, free access covered by my facility/organisation - Yes, but it is not for free - No access - Don’t know |
| On a scale of 1 (not at all) to 5 (completely), do you feel that your own concerns about the response to COVID-19 have been addressed by your facility, ward or by any professional organization to which you belong? | 1 – not at all  2 – minimally  3 – somewhat  4 – well  5 – completely  Don’t know |
| On a scale from 1 (not at all) to 4 (highly), do you consider your personal role as a health worker in this COVID-19 outbreak is **valued by the community** you are serving? | Not at all  Very little  Somewhat  Highly  Unsure/don’t know |
| At this time, what are your top 3 concerns in regard to **being able to provide care to women** **and newborns**? | [free text] |
