# Supplementary material for: Maternal and newborn healthcare providers’ work-related experiences during the COVID-19 pandemic, and their physical, psychological, and economic impacts: Findings from a global online survey
Source: PLOS Glob Public Health. 2022 Aug 5;2(8):e0000602. doi: 10.1371/journal.pgph.0000602 (PMC10021724; doi:10.1371/journal.pgph.0000602)
Supplement: S2 Appendix — (DOCX) [file pgph.0000602.s002.docx]

**S2 Appendix – Tables comparing the full sample (n=1,190) to the sub-sample excluding responses from Kazakhstan**

**Table A - Characteristics of maternal and newborn care providers**

| **Characteristic** | **Total sample n=1,191**  **n (%)** | **Without respondents from Kazakhstan**  **n=625**  **n (%)** |
| --- | --- | --- |
| **Gender** |  |  |
| Male | 242 (20.3) | 197 (31.5) |
| Female | 925 (77.7) | 413 (66.1) |
| Prefer not to say | 10 (0.8) | 7 (1.1) |
| **Job** |  |  |
| Midwife | 227 (19.1) | 158 (25.3) |
| Nurse-midwife | 66 (5.5) | 58 (9.3) |
| Nurse | 360 (30.2) | 78 (12.5) |
| Obstetrician/Gynaecologist | 249 (20.9) | 172 (27.5) |
| Neonatologist/Paediatrician | 81 (6.8) | 47 (7.5) |
| Medical doctor | 155 (13.0) | 86 (13.8) |
| Other | 38 (3.2) | 22 (3.5) |
| **Position** |  |  |
| Head of facility | 49 (4.1) | 45 (7.2) |
| Head of department or ward | 139 (11.7) | 104 (16.6) |
| Head of team | 100 (8.4) | 90 (14.4) |
| Team member | 550 (46.2) | 278 (44.5) |
| Locum or interim member | 79 (6.6) | 7 (1.1) |
| Independent or self-practicing | 124 (10.4) | 73 (11.7) |
| Other | 100 (8.4) | 11 (1.8) |
| **Facility type** |  |  |
| Public (national) | 595 (50.0) | 214 (34.2) |
| Public (university or teaching) | 116 (9.7) | 116 (18.6) |
| Public (district level or below) | 181 (15.2) | 91 (14.6) |
| Social security | 2 (0.2) | 2 (0.3) |
| Health insurance or HMO | 40 (3.4) | 4 (0.6) |
| Private | 68 (5.8) | 66 (10.6) |
| Non-governmental | 18 (1.5) | 17 (2.7) |
| Faith-based or mission | 16 (1.3) | 16 (2.6) |
| Independent/self-practicing | 70 (5.9) | 64 (10.2) |
| Other | 62 (5.2) | 25 (4.0) |
| **Area type** |  |  |
| Large city | 408 (34.3) | 257 (42.1) |
| Small city | 274 (23) | 165 (26.4) |
| Town | 157 (13.2) | 98 (15.7) |
| Village/Rural area | 258 (21.7) | 69 (11.0) |
| Other | 51 (4.3) | 17 (2.7) |
| **Healthcare facility level** |  |  |
| Multiple facilities | 339 (28.5) | 232 (37.1) |
| One facility | 829 (69.6) | 378 (60.5) |
| **Healthcare facility level** |  |  |
| Referral hospital | 225 (18.9) | 218 (34.9) |
| District/regional hospital | 231 (19.4) | 138 (22.1) |
| Health centre | 98 (8.2) | 84 (13.4) |
| Polyclinic or clinic | 307 (25.8) | 32 (5.1) |
| Health post/unit or dispensary | 12 (1.0) | 6 (1.0) |
| Birth centre | 125 (10.5) | 17 (2.7) |
| Home-based care | 5 (0.4) | 4 (0.6) |
| Independent/self-practicing | 77 (6.5) | 68 (10.9) |
| Other | 83 (7) | 43 (6.9) |

**Table B – Maternal and newborn healthcare providers’ experiences with resources, infrastructure and staffing during the COVID-19 pandemic in the month preceding the survey**

|  | **Total sample n=1,191**  **n (%)** | **Without respondents from Kazakhstan; n=625**  **n (%)** |
| --- | --- | --- |
| Staffing level |  |  |
| Staffing not affected | 455 (38.2) | 309 (49.4) |
| Staffing levels decreased | 319 (26.8) | 226 (36.2) |
| Staffing levels increased | 203 (17) | 52 (8.3) |
| Don’t know | 143 (12) | 19 (3.0) |
| **Reasons for decrease in staffing level (multiple answers allowed)** | **n=319** | **n=226** |
| Change in staff rotation or shift schedule | 167 (52.4) | 111 (49.1) |
| Staff unable to reach workplace | 56 (17.6) | 47 (20.8) |
| Staff re-assigned to COVID-19 wards | 92 (28.8) | 76 (33.6) |
| Staff isolating following exposure to COVID-19 | 111 (34.8) | 95 (42.0) |
| Staff ill with COVID-19 | 84 (26.3) | 69 (30.5) |
| Staff off due to childcare | 50 (15.7) | 43 (19.0) |
| Staff off due to stress or burnout | 58 (18.2) | 54 (23.9) |
| Don’t know | 16 (5) | 7 (3.1) |
| **Ability to reach workplace** |  |  |
| I can reach my workplace easily | 815 (68.4) | 489 (78.2) |
| I can reach my workplace with some difficulty | 190 (16) | 90 (14.4) |
| It is very difficult for me to reach the workplace | 75 (6.3) | 21 (3.4) |
| It is impossible for me to reach the workplace | 36 (3) | 5 (0.8) |
| **Reasons for difficulty in reaching workplace (multiple answers allowed)** | **n=301** | **n=136** |
| Lockdown measures | 67 (17.8) | 33 (24.3) |
| Curfew or quarantine | 43 (11.4) | 19 (14.0) |
| Public transportation availability | 181 (48.1) | 63 (46.3) |
| Other | 53 (14.1) | 29 (21.3) |
| **Sufficient gloves** |  |  |
| No | 134 (11.3) | 84 (13.4) |
| Yes | 953 (80) | 510 (81.6) |
| Not required | 22 (1.8) | 12 (1.9) |
| **Sufficient N-95 masks** |  |  |
| No | 348 (29.2) | 256 (41.0) |
| Yes | 668 (56.1) | 295 (47.2) |
| Not required | 57 (4.8) | 39 (6.2) |
| **Sufficient surgical masks** |  |  |
| No | 226 (19.0) | 112 (17.9) |
| Yes | 802 (67.3) | 460 (73.6) |
| Not required | 41 (3.4) | 21 (3.4) |
| **Sufficient face/eye protection** |  |  |
| No | 323 (27.1) | 232 (37.1) |
| Yes | 712 (59.8) | 338 (54.1) |
| Not required | 49 (4.1) | 30 (4.8) |
| **Sufficient aprons** |  |  |
| No | 250 (21.0) | 166 (26.6) |
| Yes | 688 (57.8) | 376 (60.2) |
| Not required | 64 (5.4) | 33 (5.3) |
| **Sufficient PPE to change between patients** |  |  |
| No | 418 (35.1) | 312 (49.9) |
| Yes | 648 (54.4) | 263 (42.1) |
| Not required | 69 (5.8) | 36 (5.8) |
| **Faced challenges with personal protective equipment** | 341 (28.6) | 229 (36.6) |
| **Possible to get tested for COVID-19 as a health worker** |  |  |
| No | 96 (8.1) | 68 (10.9) |
| Yes, regardless of symptoms or exposure | 379 (31.8) | 151 (24.2) |
| Yes, only if exposed to COVID-19 suspected/confirmed cases | 356 (29.9) | 211 (33.8) |
| Yes, only if symptomatic | 246 (20.7) | 158 (25.3) |
| Don’t know | 76 (6.4) | 26 (4.2) |
| **Cost of test** | **n=981** | **n=557** |
| Free of charge | 787 (80.0) | 419 (75.2) |
| Paid by healthcare worker | 84 (9.0) | 44 (7.9) |
| Other | 8 (1.0) | 5 (1.0) |
| Don’t know | 95 (10.0) | 49 (8.8) |

**Table C – Maternal and newborn healthcare providers’ experiences with aggression and support from the workplace during the COVID-19 pandemic in the month preceding the survey**

|  | **Total sample n=1,191**  **n (%)** | **Without respondents from Kazakhstan**  **n=625**  **n (%)** |
| --- | --- | --- |
| **Income changes in comparison to before the pandemic (Likert scale)** |  |  |
| Substantially lower | 149 (12.5) | 101 (16.2) |
| Somewhat lower | 171 (14.4) | 130 (20.8) |
| About the same | 576 (48.4) | 326 (52.2) |
| Somewhat higher | 80 (6.7) | 35 (5.6) |
| Substantially higher | 48 (4) | 3 (0.5) |
| Don’t know | 73 (6.1) | 7 (1.1) |
| **Exposure to aggressive behaviour in the workplace** | 304 (25.5) | 159 (25.4) |
| **Type of aggressive behaviour (multiple answers allowed)** | **n=304** | **n=159** |
| Animosity or discrimination | 102 (33.6) | 56 (35.2) |
| Harassment | 33 (10.9) | 30 (18.9) |
| Verbal aggression, shouting | 164 (53.9) | 114 (71.7) |
| Intimidation / threats | 77 (25.3) | 53 (33.3) |
| Threatening gestures, including with a weapon or a dangerous object | 13 (4.3) | 6 (3.8) |
| Physical violence (including shoving, punching, kicking, etc.) | 17 (5.6) | 13 (8.2) |
| Spitting or coughing | 31 (10.2) | 8 (5.0) |
| Sexual violence | 3 (1) | 3 (1.9) |
| Self-harm | 6 (2) | 2 (1.3) |
| **Target of the aggressive behaviour (multiple answers allowed)** | **n=304** | **n=159** |
| Healthcare providers themselves | 160 (52.6) | 100 (62.9) |
| Colleagues | 126 (41.4) | 100 (62.9) |
| Family members | 13 (4.3) | 7 (4.4) |
| Friends or relatives | 14 (4.6) | 11 (6.9) |
| Patients | 63 (20.7) | 30 (18.9) |
| Aggression toward objects (desk, wall, etc) | 19 (6.3) | 14 (8.8) |
| **Perpetrator of the aggressive behaviour (multiple answers allowed; n=304)** |  |  |
| Healthcare providers themselves | 35 (11.5) | 4 (2.5) |
| Colleagues | 35 (11.5) | 27 (17.0) |
| Family members | 11 (3.6) | 5 (3.1) |
| Friends or relatives | 11 (3.6) | 8 (5.0) |
| Community member (e.g. neighbour or teacher) | 32 (10.5) | 23 (14.5) |
| Patient | 104 (34.2) | 57 (35.9) |
| Patient’s family | 115 (37.8) | 91 (57.2) |
| Stranger | 35 (11.5) | 20 (12.6) |
| Public or government official | 29 (9.5) | 24 (15.1) |
| **Access to formal mental health support** |  |  |
| No access | 375 (31.5) | 255 (40.8) |
| Available, but not free of charge | 117 (9.8) | 84 (13.4) |
| Free access covered by facility/organisation | 381 (32) | 193 (30.9) |
| Don’t know | 231 (19.4) | 76 (12.2) |
| **Concerns addressed by facility or by professional organisation (Likert scale)** |  |  |
| Not at all | 105 (8.8) | 59 (9.4) |
| Minimally | 162 (13.6) | 118 (18.9) |
| Somewhat | 335 (28.1) | 237 (37.9) |
| Well | 332 (27.9) | 150 (24.0) |
| Completely | 151 (12.7) | 36 (5.8) |

**Table D – The psychological impact of the COVID-19 pandemic on maternal and newborn healthcare providers during the month preceding the survey**

|  | **Total sample n=1,191**  **n (%)** | **Without respondents from Kazakhstan**  **n=625**  **n (%)** |
| --- | --- | --- |
| **Feeling protected in the workplace (Likert scale)** |  |  |
| Not at all | 58 (4.9) | 39 (6.2) |
| Minimal protection | 128 (10.7) | 89 (14.2) |
| Some protection | 326 (27.4) | 225 (36.0) |
| Well protected | 399 (33.5) | 198 (31.7) |
| Completely protected | 209 (17.5) | 50 (8.0) |
| **Feeling valued by community (Likert scale)** |  |  |
| Not at all | 102 (8.6) | 69 (11.0) |
| Very little | 215 (18.1) | 143 (22.9) |
| Somewhat | 427 (35.9) | 236 (37.8) |
| Highly | 269 (22.6) | 135 (21.6) |
| Unsure/don’t know | 71 (6) | 19 (3.0) |
| **Stress levels compared to beginning of outbreak** |  |  |
| Substantially lower | 76 (6.4) | 38 (6.1) |
| Somewhat lower | 174 (14.6) | 106 (17.0) |
| Same as the beginning of the outbreak | 201 (16.9) | 90 (14.4) |
| Somewhat higher | 426 (35.8) | 261 (41.8) |
| Substantially higher | 205 (17.2) | 104 (16.6) |
