# Supplementary material for: Maternal and newborn healthcare providers’ work-related experiences during the COVID-19 pandemic, and their physical, psychological, and economic impacts: Findings from a global online survey
Source: PLOS Glob Public Health. 2022 Aug 5;2(8):e0000602. doi: 10.1371/journal.pgph.0000602 (PMC10021724; doi:10.1371/journal.pgph.0000602)
Supplement: S1 Table — (DOCX) [file pgph.0000602.s003.docx]

**S1 Table – List of countries from which respondents answered the survey**

|  | **Country** | **Full sample**  **n (%)** | **Open-text**  **n (%)** |
| --- | --- | --- | --- |
| **High income countries** | Italy | 48 (4) | 20 (3.6) |
|  | Japan | 46 (3.9) | 28 (5.1) |
|  | Norway | 29 (2.4) | 12 (2.2) |
|  | Uruguay | 27 (2.3) | 22 (4.0) |
|  | Germany | 26 (2.2) | 23 (4.2) |
|  | Saudi Arabia | 20 (1.7) | 8 (1.5) |
|  | United States | 18 (1.5) | 16 (2.9) |
|  | Belgium | 15 (1.3) | 9 (1.6) |
|  | United Kingdom | 14 (1.2) | 9 (1.6) |
|  | Switzerland | 12 (1) | 9 (1.6) |
|  | Canada | 9 (0.8) | 7 (1.3) |
|  | Spain | 7 (0.6) | 5 (0.9) |
|  | France | 6 (0.5) | 4 (0.7) |
|  | Oman | 6 (0.5) | 1 (0.2) |
|  | Portugal | 5 (0.4) | 2 (0.4) |
|  | Slovakia | 5 (0.4) | 1 (0.2) |
|  | Chile | 4 (0.3) | 4 (0.7) |
|  | Sweden | 3 (0.3) | 1 (0.2) |
|  | Austria | 2 (0.2) | 2 (0.4) |
|  | Estonia | 2 (0.2) | 2 (0.4) |
|  | Ireland | 2 (0.2) | 1 (0.2) |
|  | New Zealand | 2 (0.2) | 1 (0.2) |
|  | Trinidad and Tobago | 2 (0.2) | 2 (0.4) |
|  | Australia | 1 (0.1) | 0 (0.0) |
|  | Barbados | 1 (0.1) | 1 (0.2) |
|  | Denmark | 1 (0.1) | 1 (0.2) |
|  | Greece | 1 (0.1) | 1 (0.2) |
|  | Iceland | 1 (0.1) | 1 (0.2) |
|  | Luxembourg | 1 (0.1) | 0 (0) |
|  | Netherlands | 1 (0.1) | 0 (0) |
|  | High income countries (sub-total) | 317 (26) | 193 (35) |
| **Middle income countries** | Kazakhstan | 566 (47.5) | 112 (20.3) |
|  | Nigeria | 39 (3.3) | 31 (5.6) |
|  | Cameroon | 31 (2.6) | 24 (4.4) |
|  | Morocco | 21 (1.8) | 13 (2.4) |
|  | India | 20 (1.7) | 16 (2.9) |
|  | Tanzania | 17 (1.4) | 11 (2.0) |
|  | Bolivia | 13 (1.1) | 10 (1.8) |
|  | Brazil | 10 (0.8) | 7 (1.3) |
|  | Zambia | 10 (0.8) | 10 (1.8) |
|  | Argentina | 9 (0.8) | 9 (1.6) |
|  | Kenya | 9 (0.8) | 7 (1.3) |
|  | Bangladesh | 3 (0.3) | 3 (0.5) |
|  | Benin | 3 (0.3) | 2 (0.4) |
|  | Côte d'Ivoire | 3 (0.3) | 1 (0.2) |
|  | Egypt | 3 (0.3) | 3 (0.5) |
|  | South Africa | 3 (0.3) | 3 (0.5) |
|  | Uzbekistan | 3 (0.3) | 2 (0.4) |
|  | Laos | 2 (0.2) | 1 (0.2) |
|  | Nepal | 2 (0.2) | 1 (0.2) |
|  | Nicaragua | 2 (0.2) | 2 (0.4) |
|  | Congo, Republic of the | 1 (0.1) | 1 (0.2) |
|  | Costa Rica | 1 (0.1) | 1 (0.2) |
|  | Cuba | 1 (0.1) | 0 (0) |
|  | Dominican Republic | 1 (0.1) | 1 (0.2) |
|  | Iraq | 1 (0.1) | 1 (0.2) |
|  | Kyrgyzstan | 1 (0.1) | 1 (0.2) |
|  | Lebanon | 1 (0.1) | 1 (0.2) |
|  | Myanmar (Burma) | 1 (0.1) | 1 (0.2) |
|  | Panama | 1 (0.1) | 1 (0.2) |
|  | Peru | 1 (0.1) | 1 (0.2) |
|  | Philippines | 1 (0.1) | 1 (0.2) |
|  | Romania | 1 (0.1) | 1 (0.2) |
|  | Russia | 1 (0.1) | 0 (0) |
|  | Suriname | 1 (0.1) | 1 (0.2) |
|  | Tunisia | 1 (0.1) | 0 (0) |
|  | Vietnam | 1 (0.1) | 0 (0) |
|  | Zimbabwe | 1 (0.1) | 1 (0.2) |
|  | Middle income countries (sub-total) | 786 (66) | 281 (51) |
| **Low income countries** | Democratic Republic of the Congo | 48 (4) | 45 (8.2) |
|  | Uganda | 19 (1.6) | 18 (3.3) |
|  | Guinea | 5 (0.4) | 4 (0.7) |
|  | Syria | 4 (0.3) | 0 (0) |
|  | Afghanistan | 3 (0.3) | 1 (0.2) |
|  | Ethiopia | 2 (0.2) | 2 (0.4) |
|  | Malawi | 2 (0.2) | 2 (0.4) |
|  | Mozambique | 2 (0.2) | 2 (0.4) |
|  | Rwanda | 2 (0.2) | 2 (0.4) |
|  | Somalia | 1 (0.1) | 1 (0.2) |
|  | Low income countries (sub-total) | 88 (7) | 77 (14) |
| **Total** | | **1,191 (100)** | **551 (100)** |
