# Supplementary material for: Maternal and newborn healthcare providers’ work-related experiences during the COVID-19 pandemic, and their physical, psychological, and economic impacts: Findings from a global online survey
Source: PLOS Glob Public Health. 2022 Aug 5;2(8):e0000602. doi: 10.1371/journal.pgph.0000602 (PMC10021724; doi:10.1371/journal.pgph.0000602)
Supplement: Attachment — Submitted filename: Response to reviewer comments_27 June 2022.docx [file pgph.0000602.s004.docx]

Shela Hirani, PhD, IBCLC, RN

Editor in Chief,

PLOS Global Public Health

June 27^th^, 2022

**RE:** PGPH-D-21-00830 – “Maternal and newborn healthcare providers’ work-related experiences during the COVID-19 pandemic, and their physical, psychological, and economic impacts: findings from a global online survey”

Dear Dr Hirani,

Thank you very much for your email dated 21^st^ June 2022 and the comments and suggestions of the reviewers. We have read the peer review thoroughly and have revised our paper accordingly. We feel that the paper is much improved as a result of this peer review process, and thank you for taking it to this stage.

Please find for your kind consideration the following:

1. A ‘point by point’ response to the comments and suggestions of the reviewers (below page 2). Reviewers’ comments are in bold font followed by our responses.
2. A new revised version of the manuscript marked “Revised Manuscript with track change”
3. A revised and clean version marked “Manuscript”

While hoping that these changes would meet with your favourable consideration, we hold ourselves at your disposition for further information or other changes that might be required.

Yours sincerely,

Delphin & Aline

On behalf of co-authors

**Point by point response to the reviewers’ comments and suggestions**

**RE: Manuscript ID PGPH-D-21-00830 – “Maternal and newborn healthcare providers’ work-related experiences during the COVID-19 pandemic, and their physical, psychological, and economic impacts: findings from a global online survey”**

We thank you for reviewing this paper and for the detailed comments and suggestions. Below, we provide a point-by-point response, and attached is a revised manuscript in line with the welcomed suggestions.

**Journal Requirements:**

**1. In the Funding Information you indicated that no funding was received. Please revise the Funding Information field to reflect funding received.**

**Please ensure that the funders and grant numbers match between the Financial Disclosure field and the Funding Information tab in your submission form. Note that the funders must be provided in the same order in both places as well.**

Thank you for this comment. We have revised and updated the funding information field and added the below information to the manuscript:

“This study was funded by the Institute of Tropical Medicine’s COVID-19 Pump Priming fund supported by the Flemish Government, Science & Innovation and by the Embassy of the United Kingdom in Belgium. LB is funded in part by the Research Foundation – Flanders (FWO) as part of her Senior Postdoctoral Fellowship.” “The funders had no role in study design, data collection and analysis, decision to publish, or preparation of the manuscript.”

**2. Please update your online Competing Interests statement. If you have no competing interests to declare, please state: “The authors have declared that no competing interests exist.”**

Thank you. We updated the online declaration of competing interests as well as the statement in the manuscript to reflect the above suggestion.

**3. Please review your reference list to ensure that it is complete and correct. If you have cited papers that have been retracted, please include the rationale for doing so in the manuscript text, or remove these references and replace them with relevant current references. Any changes to the reference list should be mentioned in the rebuttal letter that accompanies your revised manuscript. If you need to cite a retracted article, indicate the article’s retracted status in the References list and also include a citation and full reference for the retraction notice.**

We have reviewed the reference list accordingly. Four new references have been added (38, 58, 59 and 60) upon answering the suggestions made by reviewers. The changes in citations and the reference list were marked-up in the manuscript as tracked changes.

**Note: the line numbers refer to changes in the marked-up copy of the manuscript.**

**Reviewer #1**

**Overall, this is a well-written manuscript. The authors have systematically examined the topic of investigation. Comparison of the results across countries of different income levels is useful in mapping the areas with greater impact. With such a high number of sample size, it would have been desirable to perform some advanced statistical analysis to identify underlying associations.**

Thank you, we appreciate your review of the manuscript which helped us improve the quality of the paper. We would like to clarify that the reason behind our lack of conducting more advanced statistical analysis is related to two issues: 1) the lack of a sampling frame for online data collection which engenders a potential selection bias and lack of representativeness of our sample data; 2) the nature of the study including qualitative data which allowed us to explore certain connections in-depth using the rich open-ended responses, which, when complemented with the quantitative findings allows us to draw solid conclusions about certain associations.

**There are a few specific comments as mentioned below.**

1. **None of the findings from the qualitative data are included in abstract. Since the study claims to be a mixed-method study, the abstract needs to include both quantitative and qualitative findings.**

We thank the reviewer for this suggestion and we have edited the abstract to include qualitative findings (Lines 48-61).

2. **The term ‘frontline healthcare providers’ used in concluding sentences of abstract (Line 58) is misleading. This term also encompasses community level health workers in many countries and this study does not include all those type of health workers.**

Thank you for this observation. We have replaced ”frontline healthcare providers” with “healthcare providers” throughout the paper (e.g. Lines 67 and 165).

3. **Was the tool used piloted and validated? Were the translated versions in different languages validated? This should be part of methods and/or limitations section.**

Many thanks for this relevant comment. Given the circumstances of data collection and conducting fieldwork during the pandemic, it was not feasible to pilot and validate the questionnaire and translations. We agree with the reviewer and have added this as another limitation of the study. For more detail, please take a look at lines 466-469 of the revised manuscript. We would also like to mention that in order to collect data for this second round, we reviewed and adapted the questionnaire used to collect data in the first round, during which we received more than 1600 responses from various countries and in different languages.

**Reviewer #2**

**General Observation - It needs to be concise and crisp.**

We thank the reviewer for this observation and acknowledge that the manuscript is lengthy as a result of the mixed-methods study design. We have revised the manuscript and made cuts throughout the paper in an attempt to deliver the message concisely.

**Methods**

**Following questions/concerns needs to be addressed under methodology section**

1. **What measures have been taken for ensuring data collection quality?**

We sincerely thank the reviewer for their comment. Considering that data collection was done through an online self-administrated questionnaire (due to the lack of a better option at the time), the research team has performed data collection quality checks to the best of their ability, including:

- Programming the questionnaire on the digital data collection platform KoboToolbox, which allowed us to introduce several quality and monitoring elements in the tool, such as automated skip-patterns and answer restrictions when relevant (Lines 157-158)
- A thorough data quality verification exercise was conducted post data collection; this involved checking and removing duplicate submissions resulting from technical errors; a thorough review of open-ended responses to the “other” answer option regarding cadre of healthcare providers, in order to ensure that the sample was limited to healthcare providers (e.g. excluding submissions from administrative staff); and dropping submissions made with a high percentage of missing answers (Lines 166 - 169)
- Having open-ended questions in the data collection tool allowed us to check the consistency of responses between close- and open-ended questions from the same respondent. We consider this as a strength of our design and we have added it to the manuscript (Line 451 - 452)

2. **Description of 551 qualitative responses can be given separately, like it was given for quantitative responses. How many responses received from high-,middle- and low-income countries.**

Thank you for this suggestion. We have added the country distribution of respondents who provided qualitative data to the supplementary file S2 Table. Additionally, the distribution by country income group is mentioned in the results section (Lines 193-193).

3. **How open text responses were analyzed, manually or through software.**

The open-text responses were analyzed manually. We have clarified this in the “Data processing and analysis” section (line 180).

**Results**:

4. **What were the new themes emerged under open text response (Classification by type of respondents, A matrix can be provided).**

The findings of the analysis of qualitative data were summarised and triangulated with the quantitative data, based on the analysis framework adopted for the whole study: i.e. working conditions and physical impact, economic impact, and psychological impact. An additional theme was identified in the open-text responses through its interactions with the previous three themes: the effect on quality of care for mother and newborn which is summarised in box 1.

**Discussion**

5. **Currently dominated by findings of low-income countries, how high- and middle-income countries were affected that can also be discussed.**

Thank you for the suggestion. We have added additional insights regarding high- and middle-income countries to the discussion (line 419 - 425).

6. **Context of covid 19 pandemic during study time in high, low- and middle-income countries can also be specified, whether it was complete lock down or restrictions were imposed.**

We added a brief description of the stringency index by country income group to the discussion section as advised (Line 376-378). However we emphasize that there is a huge variability in the COVID-19 situation and restriction measures between (and often within) countries in the same income group. We acknowledge that the interpretation of the findings should be contextualised and framed by the country-level COVID-19 situation in the limitation section (Line 455 - 458).

7. **How the condition of different level of health providers during the pandemic impacted the quality of newborn care can be focused under discussion section.**

Thank you for this suggestion, we agree and we have added a discussion on the potential influence of this issue on newborn care (line 427-431).

**Reviewer #3**

**In my point of view, this manuscript will meet PLOS Global Public Health’s publication criteria with some improvement. From a statistical point of view, it would be better to show to someone who is an expert in the field.**

We thank the reviewer for their feedback and we welcome additional feedback from statistical experts.
